# Dietary Intakes and Dietary Quality during Pregnancy in Women with and without Gestational Diabetes Mellitus - A Norwegian Longitudinal Study

**DOI:** 10.3390/nu10111811

**Published:** 2018-11-20

**Authors:** Trude Elvebakk, Ingrid L. Mostad, Siv Mørkved, Kjell Å. Salvesen, Signe N. Stafne

**Affiliations:** 1 Department of Clinical and Molecular Medicine, Norwegian University of Science and Technology (NTNU), 7089 Trondheim, Norway; Trude.Elvebakk@stolav.no (T.E.); ingrid.l.mostad@ntnu.no (I.L.M.); pepe.salvesen@ntnu.no (K.Å.S.); 2 Children’s Clinic, St. Olavs Hospital, Trondheim University Hospital, 7006 Trondheim, Norway; 3 Clinic of Clinical Services, St. Olavs Hospital, Trondheim University Hospital, 7006 Trondheim, Norway; siv.morkved@ntnu.no; 4 Department of Public Health and Nursing, Norwegian University of Science and Technology (NTNU), 7089 Trondheim, Norway; 5 Department of Obstetrics and Gynecology, St. Olavs Hospital, Trondheim University Hospital, 7006 Trondheim, Norway

**Keywords:** gestational diabetes, nutrition, nutritional recommendations

## Abstract

Gestational diabetes mellitus (GDM) is associated with maternal diet, however, findings are inconsistent. The aims of the present study were to assess whether intakes of foods and beverages during pregnancy differed between women who developed GDM and non-GDM women, and to compare dietary intakes with dietary recommendations of pregnancy. This is a longitudinal study using participants of a randomized controlled trial. Women with complete measurements of a 75 g oral glucose tolerance test (OGTT) at 18–22 and 32–36 weeks gestation were included in the cohort (*n* = 702). Women were diagnosed for GDM according to the simplified International Association of Diabetes and Pregnancy Study Group criteria at 32–36 weeks (GDM women: *n* = 40; non-GDM women: *n* = 662). Dietary data (food frequency questionnaire) was collected at both time points and compared between GDM and non-GDM women. Variability in OGTT values was assessed in a general linear model. Marginal differences between GDM and non-GDM women in intakes of food groups were found. No associations were found between dietary variables and OGTT values. Not all dietary recommendations were followed in the cohort, with frequently reported alcohol consumption giving largest cause for concern. This study did not find dietary differences that could help explain why 40 women developed GDM.

## 1. Introduction

Gestational diabetes mellitus (GDM) is defined as carbohydrate intolerance resulting in hyperglycemia of variable severity with onset or first recognition during pregnancy [1]. The pathophysiology is an inadequate pancreatic β-cell compensation for the increased insulin resistance of pregnancy [1,2,3]. GDM is associated with adverse pregnancy outcomes and increased short- and long-term morbidity in both mother and child [4,5,6,7]. Known risk factors for GDM are diabetes in first degree relatives, maternal age >25 years, non-European ethnicity, previous GDM, impaired glucose tolerance pre-pregnancy, pre-pregnancy BMI ≥25 kg/m^2^, and high gestational weight gain [8].

Maternal diet before and during pregnancy could be a modifiable risk factor of GDM, but findings have been inconsistent and flaws to the current body of evidence have been pointed out [9]. Diets resembling the Mediterranean diet or the Dietary Approach to Stop Hypertension before or during pregnancy are most consistently found to reduce risk or odds of GDM [10]. These diets include higher consumption of foods with high fiber content, such as legumes, whole grain, and vegetables, lean meats, fish, and poultry, and limited intakes of processed foods and foods with high fat and/or sugar content. GDM is reported to be positively associated with a high fat intake, the consumption of ≥7 eggs/week, ≥300 mg cholesterol/day, and an intake of ≥1.1 mg/day of heme iron during pregnancy [11,12,13]. 

The aims of the present study are (1) to assess whether intakes of foods and beverages during pregnancy differ between Nordic Caucasian women with and without GDM, and (2) to compare dietary intakes with dietary recommendations of pregnancy. 

## 2. Materials and Methods

### 2.1. Study Population

This is a longitudinal study using participants of a randomized controlled trial. The study population was participants in the Training in Pregnancy trial [14], a two-armed, two-centered randomized controlled trial that aimed to investigate whether a 12-week regular exercise program during the second half of pregnancy could prevent GDM. The study sites of St. Olavs hospital and Stavanger University hospital in Norway recruited pregnant women from April 2007 to September 2009. Written information about the trial and invitation to participate were sent by mail, along with invitation to an ultrasound scan at 17–19 weeks gestation. The ultrasound scan is free of charge and routinely offered to all pregnant women in Norway. Inclusion criteria were Caucasian women aged ≥18 years with a singleton live fetus, living ≤30 min drive from either of the two hospitals. Exclusion criteria were high-risk pregnancies and/or diseases that could interfere with participation, including, but not exclusively, a history of giving birth before 34 weeks gestation, preeclampsia, serious growth retardation in the fetus, asthma, heart disease, hypertonia, renal disease, known substance abuse, and/or in the current pregnancy, placenta previa, elevated blood pressure (>140/90 ≥two measurements) before gestational week 20, and/or identified high risk for preterm labor. Women willing to participate were not compensated financially. The study was approved by the Regional Committee for Medical and Health Research Ethics (REK 4.2007.81), and procedures followed were in accordance with ethical standards of research and the Helsinki declaration. Eligible women were enrolled the following week after the ultrasound scan and met at the study sites for assessments at 18–22 (study entry) and 32–36 weeks gestation (follow-up after the exercise intervention). 

Details on the exercise program are reported elsewhere [14]. A total of 855 women were included. All women received the brochure “Nutrition in Pregnancy”, based upon the nutritional recommendations for the Norwegian population given in 2005 [15]. The brochure gives information on nutritional requirements during pregnancy and provides suggestions on how to meet nutritional needs through examples of balanced meal compositions. 

### 2.2. Outcome Measures

At both time points (week 18–22 and week 32–36) women underwent a 2-h oral glucose tolerance test (OGTT). GDM was diagnosed according to the simplified International Association of Diabetes and Pregnancy Study Groups (IADPSG) criteria [16] as fasting plasma glucose ≥5.1 mmol/L, or 2-h value ≥8.5 mmol/L, using a 75-g glucose load. Blood samples were analyzed using standard laboratory procedures at the hospitals.

Data on daily intakes of foods and beverages were collected at week 18–22 and 32–36 using a food frequency questionnaire (FFQ) developed for use in national dietary surveys of the general adult population (see supplementary file, Figure S1). The FFQ was administered before the OGTT results were available. The women were instructed to provide information about their dietary intake as it was the previous four weeks. The FFQ is quantitative, self-administered, and optical mark readable, and has been validated against the Norwegian population [17,18,19]. Three versions of the FFQ have been developed; the NORKOST 1997 version was used in the current study. It includes questions about 180 food items, including nutritional supplements. Alternatives for frequency of use varied between per day, per week, or per month, depending on the food item. Portion sizes were specified in household units, which were later computed into edible amounts of food based on standardized scales. Related food items were categorized into 15 main groups of foods and beverages with 62 subgroups. The results were compared with the Norwegian food guidelines for the general population, which give quantitative recommendations for red and processed meat, fish and shellfish, fatty fish, and fruit and vegetables combined and separately [20]. The Norwegian food guidelines [20] are in line with recommendations given by the World Cancer Research Fund [21] and the Nordic Nutrition Recommendations [22]. 

Estimations of daily intake of energy and nutrients were computed by using a food database, based on the official Norwegian Food Composition Table [23], and a software system developed at the Institute of Nutrition Research at the University of Oslo. Factors for energy percentage (E%) calculations were: 4 kcal/g for protein and carbohydrates (minus fiber), 9 kcal/g for fat, 7 kcal/g for alcohol, and 2 kcal/g for fiber. The E% results were compared with recommended ranges of energy distribution for the general population, and micronutrient results were compared with recommendations for pregnant women [22]. 

Data on pre-pregnancy weight and height, level of exercise before pregnancy, smoking, and family history of diabetes were self-reported through questionnaires. Current weights were measured at the sites at 18–22 and 32–36 weeks gestation; all women were weighed on the same scale, fasting, without shoes, and with light clothes. BMI was calculated with the formula weight (kg)/(height (m))^2^. Level of exercise before pregnancy was presented as a categorical variable (yes/no), defined as “exercised at least three times per week at moderate to high intensity, perceived strenuous or very strenuous”. 

### 2.3. Statistical Analysis

The software package IBM SPSS 22.0 (SPSS Inc., Chicago, IL, USA) was used for statistical analysis. Assumptions of normality were assessed using Kolmogorov-Smirnov and Shapiro-Wilk tests, and by visual inspections of histograms displaying the normal curve. Due to skewed dietary data and unequal variance in sample sizes, non-parametric testing of differences between groups was done for continuous data. Group medians (50th) and 25th and 75th percentiles were computed for continuous variables, which were analyzed using Mann-Whitney U-Test. Differences between women with and without GDM in intakes of foods and beverages were tested with a total of 15 tests for the main food groups and 62 tests for the food subgroups. Categorical variables were analyzed using Pearson’s Chi Square Test or Fisher’s exact test. Variability in glucose levels was assessed with one-way ANOVA (Univariate Analysis of Variance). Predictor variables in the model were selected based on correlations with glucose variables, assessed with Pearson’s correlation coefficient and Spearman rank correlation coefficient. Two-sided significance was assumed at the 5% level (*p* ≤ 0.05). 

## 3. Results

### 3.1. Study Population

**Figure 1:**
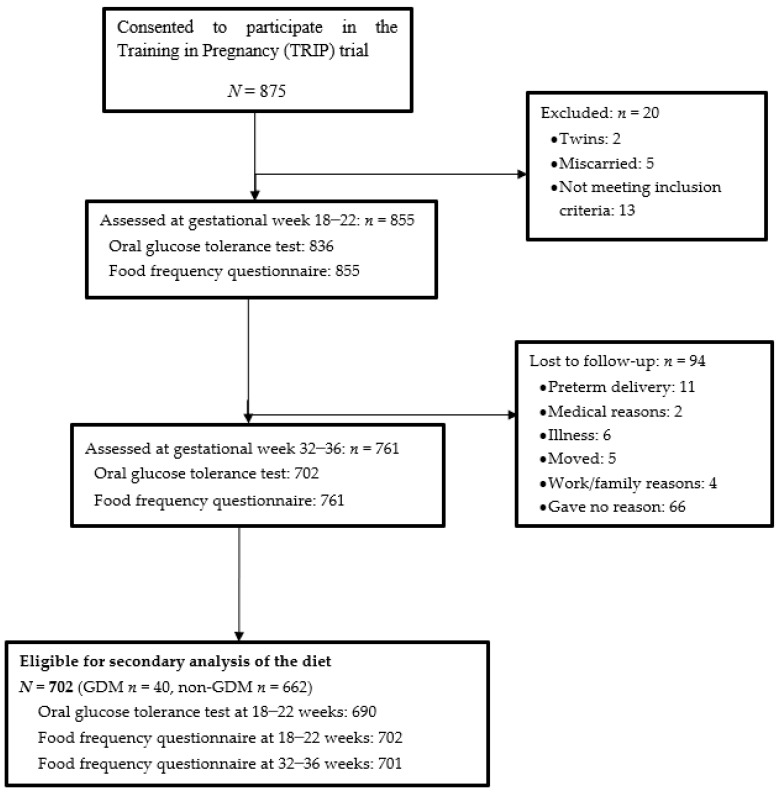
Flow chart of study participants.

Approximately 12,000 pregnant women had routine ultrasound scans at St. Olavs hospital and Stavanger University hospital between 2007 and 2009, whereof 875 (7%) consented to participate. A total of 855 women met the inclusion criteria. The current study includes all women who completed the OGTT at both time points, resulting in a cohort of 702 women (Figure 1). Women were analyzed as cases (GDM women; *n* = 40) or controls (non-GDM women; *n* = 662), as diagnosed after the OGTT at 32–36 weeks. 

GDM women had higher pre-pregnancy weight (67 vs. 64 kg, *p* = 0.028) and pre-pregnancy BMI (24.2 vs. 22.5, *p* = 0.009), and gained more weight during pregnancy compared with non-GDM women (5.4 vs. 4.5 kg, *p* = 0.025 and 14.3 vs. 11.8 kg, *p* = 0.012 at 18–22 and 32–36 weeks gestation, respectively) (Table 1). Fifteen GDM women (38%) had a pre-pregnancy BMI ≥25 compared with 129 (20%) non-GDM women (*p* = 0.014). Further, both fasting glucose and 2-h glucose were higher in GDM women at 18–22 weeks gestation, and a higher proportion of GDM women had a family history of diabetes in first-degree relatives (Table 1). There were no differences between the two groups in maternal age at study entry, height, self-reported exercise before pregnancy, smoking, allocation, or protocol adherence in the trial (Table 1). 

### 3.2. Intakes of Foods and Beverages during Pregnancy

There were no differences between GDM and non-GDM women in intake of the 15 main groups of foods and beverages at either time point during pregnancy (Table 2). Regarding food subgroups, GDM women reported lower intake of whey cheese (0 vs. 10 g/day, *p* = 0.009) in week 18–22, higher intake of French fries (7 vs. 3 g/day, *p* = 0.042) in week 32–36, and lower intake of fruit juices at both time points (62 vs. 108 g/day, *p* = 0.027, and 54 vs. 107 g/day, *p* = 0.010, in 18–22 and 32–36 weeks of pregnancy, respectively) (Table 2). 

### 3.3. Intakes of Energy and Nutrients during Pregnancy

Energy intake, fiber intake, and energy distribution did not differ between GDM and non-GDM women at any time point during pregnancy (Table 3). Regarding micronutrients, GDM women had higher estimates of thiamin (1.7 vs. 1.5 mg/day, *p* = 0.008) and riboflavin (2.0 vs. 1.8 mg/day, *p* = 0.028) at pregnancy week 32–36. Estimates of micronutrients did not differ between GDM and non-GDM women at 18–22 weeks (Table 3). 

### 3.4. Dietary Intakes in Relation to Dietary Recommendations

Intakes of red and processed meat, fish and shellfish, and fruit and vegetables combined and separately did not differ between GDM and non-GDM women at any time point, except for a lower intake of fatty fish among the GDM women at pregnancy week 32–36 (28 vs. 57 g/week, *p* = 0.014) (Table 3). For both groups, the weekly intake of red and processed meat exceeded the recommendation of ≤500 g/week, and intakes of vegetables and fatty fish were below the recommendations of ≥250 g/day (vegetables) and 200 g/week (fatty fish). Further, both groups reported an intake of fish and shellfish close to the lowest recommendation of intake (300 g/week) and met the recommendation of a daily intake of fruit and vegetables combined of ≥500 g/day.

Energy distribution was equal and within the recommended ranges for both groups at both time points, even the limit of <10 E% from sugar was kept. Further, fiber intake was within the recommendation of 25–35 g/day. Most women reported zero intake of alcohol (Table 3). However, 243 (35%) and 246 (35%) women reported drinking light beer/lager at 18–22 and 32–36 weeks, respectively. Also, 35 (5%) and 21 (3%) reported drinking wine, and 5 (1%) and 1 (<1%) reported drinking liquor at 18–22 and 32–36 weeks, respectively. At 18–22 weeks, the median intakes were 35 g/day (25th, 75th percentiles: 14, 49 g/day) for light beer/lager, 5 g/day (1, 17 g/day) for wine, and 6 g/day (4, 6 g/day) for liquor, among women who reported intake of those beverages. At 32–36 weeks, the respective median intakes were 25 g/day (14, 49 g/day) for light beer/lager, 5 g/day (1, 15 g/day) for wine, and 6 g/day for liquor. Among those women who reported alcohol consumption (35%), the intake of pure alcohol was 0.5 g/day (0.2, 0.7 g/day) at 18–22 weeks, and 0.6 g/day (0.2, 0.6 g/day) at 32–36 weeks. In Norway, the alcohol content of light beer is 0.7–2.7%, for lager is 2.7–4.7%, and for wine is 8–14% (red, rosé, white wines). Alcohol-free beers are not included in the light beer/lager variable.

Intake of folate and iron were too low according to the recommendations for all women at both time points. GDM women met the vitamin D recommendations at 32–36 weeks gestation, whereas non-GDM women had intakes lower than the recommendation at both time points (Table 3). In all, 122 (17%) and 46 (7%) women met the recommendations and had intakes of ≥10 µg/day of vitamin D, ≥400 µg/day of folate, and ≥ 15 mg/day for iron at 18–22 and 32–36 weeks, respectively. Daily intake of other micronutrients was in accordance with recommendations at both time points (Table 3). 

### 3.5. Variability in Glucose Concentrations

Correlations between fasting and 2-h glucose values (both time points) and dietary variables (both time points) and known GDM risk factors were assessed (see supplementary material, Tables S1–4). The correlations were weak and the effect differences minor. 

Variables correlating with glucose values (fasting and 2-h) were analyzed with one-way ANOVA in order to quantify linear relationships. In all, four one-way ANOVA models were made for both fasting and 2-h glucose at both 18–22 and at 32–36 weeks gestation (see supplementary materials, Tables S5–8). At 32–36 weeks, linear relationships were found between pre-pregnancy BMI, body weight (both pre-pregnancy and at 18–22 weeks), and fasting glucose (see supplementary materials, Table S7). For the 32–36 week 2-h value, linearity was found with maternal age, pre-pregnancy BMI, and not exercising before pregnancy, with the highest β-coefficient found for not exercising before pregnancy (see supplementary materials, Table S8). The correlations between dietary variables and both fasting and 2-h glucose disappeared when pre-pregnancy BMI, weight variables, maternal age, and pre-pregnancy exercising were controlled for (see supplementary materials, Tables S5–8). 

## 4. Discussion

Only marginal differences between GDM and non-GDM women in dietary intakes were found. However, not all dietary recommendations were followed, with too low intakes of vitamin D, folate, and iron, and frequently reported alcohol consumption, giving the largest cause for concern. GDM women had higher gestational weight gain and known pre-pregnancy risk factors (overweight and maternal age) were confirmed in the present population. 

The few dietary differences between GDM and non-GDM women could have been due to chance. The statistical analysis of dietary data included many variables, and multiple testing was not accounted for. Thus, with a significance level of 0.05, one expects three tests of the food subgroups and one test of the micronutrients to be significant due to chance per time point for testing. 

The differences between GDM and non-GDM women in intakes of fruit juices, whey cheese, and French fries did not generate differences in the main food groups or alter energy distribution or total energy intake between groups. Therefore, it is uncertain whether the different consumption levels by GDM women of these food subgroups is of clinical significance. As prevalence of overweight was low in the present study, we could speculate the possibility that β-cell insufficiency and not insulin resistance was a plausible primary cause of GDM in these women. If the women had been more overweight, insulin resistance could have been more pronounced and dietary factors could have had greater impact on blood glucose concentrations. 

The reported diet of the present population was not optimal according to dietary recommendations [16,18], as an excess intake of red and processed meat, along with low intakes of vegetables and fatty fish, were observed for all women, GDM or not. This is supported by findings from the Norwegian Mother and Child Cohort Study (MoBa), where only 45% of the pregnant women followed the recommendation for red and processed meat, and <10% followed the recommendations for vegetables and fatty fish [24]. When evaluating the whole diet of a group, one must be aware that no clear cut-off values for intakes of food groups is established regarding risk of chronic diseases, and that individual dietary needs may differ from recommended ranges of intake. All women in the present study were given written information on food guidelines during pregnancy available at that time. However, quantitative recommendations of food groups were not included in the food guidelines until 2011 [17]. 

Despite supplementation being included in the nutrient estimates, too low intakes of vitamin D, folate, and iron were observed for women in both groups, with the exception of adequate vitamin D intake in GDM women at 32–36 weeks. The low reported intake of vitamin D has recently been verified by serum analyses in the present population, as 34% had low vitamin D serum levels (25(OH)D <50 nmol/L) at 32–36 weeks gestation [25]. Similar results have been observed in Sweden [26], where dietary vitamin D intakes <10 µg/day in fair-skinned pregnant women were found, and most women were vitamin D deficient by their third trimester. Norway and Sweden are comparable regarding diet, latitude, and economy and the large number of pregnant women with low vitamin D levels raise concern. Folate supplementation of 400 µg/day before pregnancy and in the first trimester is recommended [27]. Dietary data was collected at the second and third trimester, which could explain folate estimates below recommended levels. However, data from the Medical Birth Registry of Norway show that only 33% and 79% of women took folate supplements before and during pregnancy, respectively [28]. It is difficult to evaluate whether women met their iron needs, as iron needs during pregnancy are dependent upon iron stores at conception. Considering that this cohort consisted of healthy Caucasian women, dietary inadequacies of greater severity may possibly be found in the general pregnant population.

Surprisingly, 35% of women reported alcohol consumption during pregnancy. A similar proportion of pregnant women reported alcohol consumption in a study from Oslo, Norway [29]. With Trondheim, Stavanger, and Oslo being three of the five largest cities in Norway, this may indicate that a significant proportion of urban Norwegian women do not abstain from alcohol while pregnant, despite total abstinence being strongly recommended [17]. Light beer/lager beer were the most frequently reported alcoholic beverages in the present study, followed by wine and liquor. Interestingly, in public surveys of the general population, beer only accounts for 25% of the alcohol use among Norwegian women, while wine accounts for >60% [30]. The different alcohol pattern observed in the present study versus the general female population could indicate that pregnant women may perceive beer as less harmful to the fetus than wine or liquor.

Advanced maternal age and high pre-pregnancy BMI are known to increase the risk of elevated OGTT values [31], as supported by our results. Regular exercise before pregnancy is found to decrease GDM risk in observational studies [32], corresponding to our results of a negative linear relationship between the 2-h glucose value at 32–36 weeks and pre-pregnancy exercise. Neither food groups nor nutrients were associated with glucose values in the present study, which is in line with findings from Project Viva [33]. Apart from the non-modifiable risk factor of maternal age, the present results mainly show a relationship with glucose values for behavior before rather than during pregnancy. Findings from the Nurses’ Health Study II also suggest that a healthy lifestyle before pregnancy is protective against GDM, with a risk reduction of 52% for normal weight, physically active, non-smoking women with a healthy diet [34]. The associations with glucose values were weak in the present study, however, with increasing obesity rates [35] and age [36] among Norwegian primiparous women, the higher pre-pregnancy weight and BMI, and higher gestational weight gain in GDM women are of concern to public health. 

The presentation of dietary data as food groups and nutrients is the primary strength of the present study, as it gives comprehensive information about the whole diet of a selected group of pregnant women. Collecting dietary data before OGTT results were available limits the possibility of social-desirability bias affecting dietary reports. Further, this study used validated assessment methods. The two-centered design and confirmation of known GDM risk factors increases the generalizability of our findings. Limitations to the present study are related to the study design for the primarily analysis [14]. The World Health Organization’s diagnostic criteria (fasting glucose ≥7.0 mmol/L or 2-hour value ≥7.8 mmol/L) [37] for GDM was the standard diagnostic criteria in Norway at the time when this study was initiated and conducted, which is why we do not have data on 1-h glucose. Even though the 1-h glucose value is missing, we believe that diagnosing according to the simplified IADPSG criteria [16] enhances the scientific value of our paper. Not grouping the women according to the original randomized allocation increases the risk of selection bias. FFQs have been criticized as being a dietary assessment method of low precision [38,39]. Since dietary assessment was not the primary aim [14], the dietary assessment method needed to provide low respondent burden, be low-cost, and easy to administer.

The total number of eligible pregnant women served by St. Olavs hospital and Stavanger University hospital between 2007 and 2009 is not known, however, the present study included less than 10% of the women attending ultrasounds during that period. In comparison, MoBa had included 42% of all eligible women in 2009 [40]. Because the MoBa study population had characteristics similar to the present population, we find the external validity of the present study acceptable for Norway. Caution is still advised regarding the generalization of these findings to overweight and ethnic diverge pregnant populations. 

## 5. Conclusions

In conclusion, this study did not find dietary differences that could help explain why 40 healthy Caucasian women developed GDM. We suggest that measures must be taken to ensure adequate nutrition and abstinence from alcohol in pregnant women. 

## Figures and Tables

**Table 1:** Characteristics of the Study Population.

	GDM Women *n* = 40			Non-GDM Women *n* = 662			
	25th	Median	75th	25th	Median	75th	*p* *
**Pre-pregnancy**							
Height (m)	1.65	1.69	1.72	1.64	1.68	1.73	0.495
Weight (kg)	61	67	74	59	64	70	0.028
BMI (kg/m^2^)	21.6	24.2	27.0	21.0	22.5	24.3	0.009
Exercised regularly ^†^ *n* (%)		12 (30)			220 (33)		0.732
**Gestational week 18–22**							
Age (year)	27	32	35	28	30	33	0.281
Weight (kg)	66.3	73.9	81.5	63.5	68.4	74.7	0.003
Weight gained (kg)	3.7	5.4	7.9	2.9	4.5	6.3	0.025
OGTT glucose level **^‡^** (mmol/L)							
Fasting	4.4	4.7	5.0	4.1	4.3	4.5	<0.001
2-h	4.8	5.6	6.3	4.2	4.8	5.4	<0.001
Had one or more children before *n* (%)	20 (50)			276 (42)			0.325
Smoking *n* (%)	1 (3)			5 (1)			0.298
Diabetes in first degree-relatives *n* (%)							
Yes	3 (8)			58 (9)			0.024
No	30 (84)			570 (90)			
Do not know	3 (8)			6 (1)			
Allocated to intervention group *n* (%)	17 (43)			358 (54)			0.191
Adhered to the exercise protocol *n* (%)	9 (28)			203 (40)			0.197
**Gestational week 32–36**							
Weight (kg)	73.2	81.8	89.4	70.0	75.9	82.3	0.001
Weight gained (kg)	10.8	14.3	17.6	9.0	11.8	13.9	0.012
OGTT glucose level **^‡^** (mmol/L)							
Fasting	4.9	5.2	5.4	4.0	4.2	4.5	<0.001
2-h	6.0	7.2	9.0	4.8	5.5	6.3	<0.001
Smoking *n* (%)		0 (0)			4 (1)		1.00

* Analysis is performed with Mann-Whitney U-test for continuous data, and Pearson’s Chi Square or Fisher’s Exact Test for categorical data. ^†^ Exercised regularly was defined as “exercising three times per week or more at moderate to high intensity”. ^‡^ OGTT, oral glucose tolerance test. GDM, Gestational diabetes mellitus.

**Table 2:** Daily intakes of main groups of foods and beverages during pregnancy, with selected subgroups.

	Gestational Week 18–22							Gestational Week 32–36						
	GDM Women *n* = 40			Non-GDM Women *n* = 662				GDM Women *n* = 40			Non-GDM Women *n* = 661			
Edible Amounts, g/day	25th	Median	75th	25th	Median	75th	*p* *	25th	Median	75th	25th	Median	75th	*p* *
Bread	146	189	242	133	174	217	0.198	124	175	218	134	168	207	0.762
Cereals	40	60	89	39	61	86	0.844	42	63	103	38	59	87	0.294
Cakes	9	17	31	10	19	31	0.496	13	21	37	12	21	33	0.641
Potatoes	20	37	61	24	41	61	0.791	22	48	61	21	34	58	0.652
French fries	0	3	10	1	3	10	0.965	1	7	11	1	3	7	0.042
Vegetables	150	233	284	127	200	296	0.303	105	177	271	117	177	256	0.898
Fruit and berries	191	296	382	229	322	448	0.343	211	275	397	222	329	479	0.729
Fruit juices	34	62	140	54	108	157	0.027	23	54	108	47	107	161	0.010
Meat	64	96	139	77	104	137	0.610	65	93	127	71	98	126	0.611
Fish and shellfish	28	41	61	29	48	71	0.479	25	38	58	27	43	67	0.266
Eggs	9	18	28	9	17	22	0.378	10	17	22	8	14	20	0.190
Dairy products ^†^	210	367	502	176	341	514	0.484	319	405	515	202	363	554	0.287
Cheese	10	23	52	15	32	51	0.252	13	32	52	14	29	45	0.627
Whey cheese	0	0	5	4	10	24	0.009	0	2	10	0	5	10	0.140
Butter, margarine, oil	19	34	47	15	29	45	0.135	19	33	51	15	28	42	0.089
Sugar and sweets	11	21	37	10	17	28	0.152	13	19	31	11	19	30	0.681
Beverages ^‡^	773	915	1531	728	1028	1510	0.925	779	1080	1551	658	957	1341	0.055
Various intake ^§^	82	122	187	77	114	164	0.332	73	107	161	74	106	150	0.728

* Differences between groups analyzed with Mann-Whitney U-test. ^†^ The “Dairy products” variable mainly includes milk and yoghurt. ^‡^ The “Beverages” variable includes water, coffee, tea, mineral water, and alcoholic beverages. ^§^ The “various intake” variable includes salty snacks, potato chips, nuts, and various other products that do not fit into the other categories above.

**Table 3:** Intake of food groups, energy, and nutrients, including supplements, in relation to recommendations.

		Gestational Week 18–22							Gestational Week 32–36						
	Recommendations	GDM Women *n* = 40			Non-GDM Women *n* = 662				GDM Women *n* = 40			Non-GDM Women *n* = 661			
		25th	Median	75th	25th	Median	75th	*p* *	25th	Median	75th	25th	Median	75th	*p* *
**Food groups, per day or week**															
Fruit and vegetables g/day	≥500 ^†^ g/day	391	542	652	404	531	710	0.430	363	494	670	376	521	712	0.604
Vegetables g/day	≥250 ^†^ g/day	150	233	284	127	200	296	0.303	105	177	271	117	177	256	0.898
Red and processed meat g/week	≤500 ^†^ g/week	423	588	964	462	646	864	0.771	383	590	842	438	613	808	0.721
Fish and shellfish g/week	300–450 ^†^ g/week	198	288	427	204	337	498	0.479	174	263	403	189	298	468	0.266
Fatty fish g/week	200 ^†^ g/week	8	49	116	25	64	123	0.157	0	28	83	17	57	116	0.014
**Energy and macronutrients per day**															
Total energy intake kcal		1963	2164	2561	1755	2086	2451	0.238	1826	2213	2605	1741	2060	2421	0.208
Total fat E%	25–40 E% ^‡^	29	34	37	29	33	36	0.690	29	33	37	29	33	36	0.587
Protein E%	10–20 E% ^‡^	15	16	17	15	16	17	0.962	14	15	17	14	16	20	0.456
Carbohydrates E%	45–60 E% ^‡^	45	49	52	45	48	51	0.796	45	50	53	46	49	52	0.880
Added sugar E%	<10 E% ^‡^	4	7	9	5	6	9	0.548	6	6	9	5	7	9	0.962
Alcohol E%	Abstinence ^§^	0	0	0.1	0	0	0.1	0.930	0	0	0.1	0	0	0.1	0.612
Fiber g	25–35 g ^‡^	25	28	33	23	28	33	0.453	23	27	36	22	27	33	0.600
**Micronutrients per day**															
Cholesterol (mg/mL)		193	274	331	205	266	325	0.937	205	255	288	197	252	312	0.867
Vitamin A RAE ^‖^	800 µg ^¶^	839	1386	1686	879	1195	1617	0.640	744	1162	1258	838	1142	1542	0.925
Vitamin D (µg)	10 µg ^¶^	6.0	9.2	13.7	5.5	8.6	14.1	0.712	7.0	10.2	14.0	5.2	8.3	13.9	0.166
Tocopherol (mg)	10 µg ^¶^	10.4	17.5	22.2	11.7	16.7	22.6	0.955	11.8	17.8	25.4	10.8	16.4	22.0	0.220
Thiamin (mg)	1.5 mg ^¶^	1.4	1.6	2.3	1.3	1.6	2.0	0.204	1.4	1.7	2.4	1.2	1.5	2.0	0.008
Riboflavin (mg)	1.6 mg ^¶^	1.6	1.9	2.6	1.4	1.9	2.4	0.329	1.6	2.0	2.9	1.4	1.8	2.4	0.028
Folate (µg)	400 µg ^#^	244	314	581	232	313	453	0.515	223	299	443	209	270	362	0.062
Vitamin C (mg)	85 mg ^¶^	111	164	220	110	153	217	0.548	106	158	252	99	145	202	0.241
Calcium (mg)	900 mg ^¶^	758	930	1260	718	919	1154	0.481	853	1003	1239	711	932	1155	0.068
Fe (mg)	15 mg **	9.0	11.0	13.1	8.9	10.9	14.0	0.932	8.5	11.0	18.6	8.6	10.7	14.2	0.236
Magnesium (mg)	280 mg ^¶^	301	354	403	288	341	410	0.542	308	359	431	285	342	408	0.867

* Differences between groups analyzed with Mann-Whitney U-test. ^†^ Norwegian Council of Nutrition Recommendation of 2011. ^‡^ Nordic Nutrition Recommendations (NNR) of 2012. ^§^ NNR (2012), reference applies to pregnant women. ^‖^ Retinol activity equivalents (RAE); 1 RAE: 1 µg retinol = 12 µg β-carotene. ^¶^ NNR (2012), references apply to pregnant and breastfeeding women. ^#^ NNR (2012), reference applies to women of reproductive age. ** NNR (2012), reference applies to women in age groups 18–30 and 31–60 years.

## Supplementary Materials

The following is available online at http://www.mdpi.com/2072-6643/10/11/1811/s1, Figure S1: Food frequency questionnaire (in Norwegian); Tables S1–4: Variables showing correlations with glucose values; Tables S5–8: Variables showing linear relationships with glucose values.
