# Supplementary material for: Dietary Intakes and Dietary Quality during Pregnancy in Women with and without Gestational Diabetes Mellitus - A Norwegian Longitudinal Study
Source: Nutrients. 2018 Nov 20;10(11):1811. doi: 10.3390/nu10111811 (PMC6266178; doi:10.3390/nu10111811)
Supplement: Supplementary file 1 [file nutrients-10-01811-s001.pdf]

Table S1. Variables showing correlations with 18–22 week fasting glucose.

	Correlation Coefficient	*p*-Value*
18–22 week whey cheese	−0.086	0.023
Pre-pregnancy BMI	0.281	<0.001
Pre-pregnancy weight	0.243	<0.001
18–22 week weight	0.260	<0.001
Maternal age at study entry	0.210	<0.001

*Analyzed with Pearson's Correlation Coefficient for normally distributed data and Spearman rank correlation coefficient for skewed data.

Table S2. Variables showing correlations with 18–22 week 2-hour glucose.

	Correlation Coefficient	*p*-Value*
18–22 week cereals	−0.088	0.011
18–22 week eggs	−0.092	0.016
18–22 week whey cheese	−0.088	0.020
18–22 week fat (g/day)	−0.076	0.046
18–22 week cholesterol	−0–112	0.003
18–22 week vitamin D	−0–111	0.004
Pre-pregnancy weight	0.155	<0.001
Pre-pregnancy BMI	0.242	<0.001
18–22 week weight	0.183	<0.001
Maternal age at study entry	0.107	0.006

*Analyzed with Pearson's Correlation Coefficient for normally distributed data and Spearman rank correlation coefficient for skewed data.

Table S3. Variables showing correlations with 32–36 week fasting glucose.

	Correlation Coefficient	*p*-Value*
18–22 week dairy products	−0.084	0.026
18–22 week cheese	−0.084	0.026
18–22 week beverages	0.080	0.035
18–22 week whey cheese	−0.144	<0.001
18–22 week milk and yoghurt	−0.086	0.022
18–22 week protein (g/d)	−0.080	0.088
32–36 week dairy products	−0.085	0.025
32–36 week French fries	0.114	0.002
32–36 week milk and yoghurt	−0.093	0.014
32–36 week cream/sour cream/ice cream	0.095	0.012
Pre-pregnancy BMI	0.267	<0.001
Pre-pregnancy weight	0.267	<0.001
18–22 week weight	0.285	<0.001
32–36 week weight	0.260	<0.001
Maternal age at study entry	0.095	0.013

*Analyzed with Pearson's Correlation Coefficient for normally distributed data and Spearman rank correlation coefficient for skewed data.

Table S4. Variables showing correlations with 32–36 week 2-hour glucose.

	Correlation Coefficient	*p*-Value*
Maternal age at study entry	0.157	<0.001
Pre-pregnancy BMI	0.161	<0.001
Pre-pregnancy exercising	−0.086	0.023

*Analyzed with Pearson's Correlation Coefficient for normally distributed data and Spearman rank correlation coefficient for skewed data.

Table S5. Variables showing linear relationships with 18–22 weeks fasting glucose (N = 702), analyzed with one-way ANOVA.

		B* (95% CI)	*p*-Value
18–22 week whey cheese	N = 702	−0.001 (−0.003, 0.001)	0.295
Pre-pregnancy BMI	N = 699	0.034 (0.017, 0.051)	<0.001
Pre-pregnancy weight	N = 699	−0.010 (−0.020, 0.000)	0.057
18–22 week weight	N = 700	0.009 (0.000, 0.018)	0.056
Maternal age at study entry	N = 702	0.016 (0.010, 0.023)	<0.001

* β = βeta coefficient.

Table S6. Variables showing linear relationships with 18–22 weeks 2-hour glucose (N = 702), analyzed with one-way ANOVA.

		B* (95% CI)	*p*-Value
18–22 week cereals	N = 702	0.000 (−0.002, 0.002)	0.695
18–22 week eggs	N = 702	−0.002 (−0.013, 0.008)	0.672
18–22 week whey cheese	N = 702	−0.004 (−0.011, 0.002)	0.172
18–22 week fat (g/d)	N = 702	0.001 (−0.004, 0.006)	0.652
18–22 week cholesterol	N = 702	−0.001 (−0.003, 0.001)	0.569
18–22 week vitamin D	N = 702	−0.006 (−0.018, 0.005)	0.282
Pre-pregnancy weight	N = 699	−0.054 (−0.085, −0.024)	0.001
Pre-pregnancy BMI	N = 699	0.142 (0.092, 0.193)	<0.001
18–22 week weight	N = 700	0.031 (0.004, 0.057)	0.022
Maternal age at study entry	N = 702	0.019 (0.001, 0.037)	0.035

* β = βeta coefficient.

Table S7. Variables showing linear relationships with 32–36 weeks fasting glucose (N = 702), analyzed with one-way ANOVA.

		B* (95% CI)	*p*-Value
18–22 week dairy products	N = 702	−0.001 (−0.003, 0.001)	0.366
18–22 week cheese	N = 702	−0.001 (−0.002, 0.001)	0.387
18–22 week beverages	N = 702	9.975E−6 (−4.151E−5, 6.156E−5)	0.704
18–22 week whey cheese	N = 702	−0.002 (−0.004, 0.001)	0.287
18–22 week milk and yoghurt	N = 702	0.001 (−0.001, 0.002)	0.393
18–22 week protein (g/d)	N = 702	0.000 (−0.002, 0.002)	0.829
32–36 week dairy products	N = 701	0.113 (−0.456, 0.682)	0.696
32–36 week French fries	N = 702	0.003 (−0.001, 0.007)	0.097
32–36 week milk and yoghurt	N = 702	−0.113 (−0.682, 0.455)	0.696
32–36 week cream/sour cream/ice cream	N = 702	−0.113 (-0.681, 0.456)	0.697
Pre-pregnancy BMI	N = 699	0.032 (0.012, 0.052)	0.002
Pre-pregnancy weight	N = 699	−0.014 (−0.026, −0.002)	0.026
18–22 week weight	N = 700	0.023 (0.007, 0.040)	0.006
32–36 week weight	N = 699	−0.007 (−0.019, 0.005)	0.238
Maternal age at study entry	N = 702	0.007 (−0.001, 0.014)	0.074

* β = βeta coefficient.

Table S8. Variables showing linear relationships with 32–36 weeks 2-hour glucose (N = 702), analyzed with one-way ANOVA.

		B* (95% CI)	*p*-Value
Maternal age at study entry	N = 702	0.042 (0.021, 0.063)	<0.001
Pre-pregnancy BMI	N = 699	0.065 (0.035, 0.094)	<0.001
Pre-pregnancy exercising (= no)	N = 457	0.193 (0.005, 0.381)	0.045

* β = βeta coefficient.
